# Acquired von Willebrand syndrome in young children with congenital heart defects: focus on patent ductus arteriosus and ventricular septal defect

**DOI:** 10.1016/j.rpth.2025.102995

**Published:** 2025-08-07

**Authors:** Oksana Trębacz, Katarzyna Szafarz, Joanna Zdziarska, Jacek Podlewski, Piotr Weryński, Wojciech Tarała, Teresa Iwaniec

**Affiliations:** 1 Department of Pediatrics and Pediatric Gastroenterology with Pediatric Cardiology Subdivision, St. Jadwiga the Queen Clinical Regional Hospital No. 2, Rzeszow, Poland; 2 Department of Neurobiology, Maj Institute of Pharmacology Polish Academy of Science, Krakow, Poland; 3 Department of Hematology and Internal Medicine, University Hospital, Krakow, Poland; 4 Dover Fueling Solutions, Krakow, Poland; 5 Department of Haematology, Jagiellonian University Medical College, Krakow, Poland

**Keywords:** acquired von Willebrand syndrome, congenital heart defect, multimer analysis

## Abstract

**Background:**

Acquired von Willebrand syndrome (AVWS) is a rare bleeding disorder due to a deficiency of von Willebrand factor (VWF). High shear stress causes stretching and rupture of VWF multimers, leading to functional loss and increased proteolysis. This occurs in cardiovascular diseases, reducing high-molecular-weight multimers (HMWMs). Patent ductus arteriosus (PDA) and ventricular septal defect (VSD) cause blood shunting between systemic and pulmonary circulation, increasing shear stress, and may contribute to AVWS.

**Objectives:**

To investigate whether children with PDA and VSD experience disturbances in platelet-related activity that cause HMWM loss and an AVWS-like phenotype.

**Methods:**

The study involved 54 children with PDA and VSD. Patients who met the screening criteria, including a ristocetin cofactor activity to VWF antigen ratio (VWF:RCo/VWF:Ag) and/or collagen binding to VWF:Ag ratio (VWF:CB/VWF:Ag) <0.8, underwent VWF multimer analysis, and the VWF large multimer index (VWF-LMI) was calculated.

**Results:**

Of the 54 patients, 26 (48.1%) underwent multimer analysis, and an AVWS-like phenotype was found in 13 (24.1%). These patients had significantly lower percentage of HMWMs and lower VWF-LMI (27.3 ± 2.9% vs 38.8 ± 5.5% and 75.5 ± 7.3 vs 108.1 ± 14.7, respectively, *P* < .001). A VWF-LMI <0.8 effectively predicted an AVWS-like phenotype with a sensitivity of 1.0 and a specificity of 0.87, followed by the VWF:CB/VWF:Ag ratio, with a sensitivity of 0.57 and specificity of 0.80 at the same threshold.

**Conclusion:**

Nearly a quarter (25%) of children with VSD and PDA exhibit an AVWS-like phenotype. In addition to, VWF multimer analysis and VWF-LMI assessment, the VWF: CB/VWF:Ag ratio is suitable for screening in this group.

## Essentials

• The prevalence of AVWS in certain heart defects is unknown.
• High shear stress in heart defects leads to loss of function of VWF.
• Almost 25% of children with some congenital heart defects exhibit an AVWS-like phenotype.
• The VWF collagen binding activity to antigen ratio and the large multimer index are predictors of AVWS.

## 1 Introduction

Acquired von Willebrand syndrome (AVWS) is a rare bleeding disorder characterized by either quantitative or qualitative deficiencies in von Willebrand factor (VWF) [1]. The causes of these deficiencies are varied and include a spectrum of diseases, such as lymphoproliferative, myeloproliferative, cardiovascular, and other conditions. The pathophysiology of AVWS is dependent on the underlying disease process responsible for the VWF defect. For example, the enhanced clearance of VWF may result from the binding of autoantibodies, which is characteristic of lymphoproliferative disorders, adsorption into malignant cells, or increased proteolysis due to elevated shear stress in cardiovascular conditions [2]. High shear stress is known to stimulate the stretching and rupture of VWF multimers, resulting in a loss of their function and making them susceptible to proteolysis by ADAMTS-13 (a disintegrin and metalloproteinase with a thrombospondin type 1 motif, member 13), a metalloprotease enzyme that cleaves large VWF multimers [3]. Thus, the accelerated clearance of VWF from circulation while maintaining normal synthesis is characteristic of AVWS in cardiovascular disorders. Consequently, the loss of high-molecular-weight multimers (HMWMs) is typical of AVWS and serves as the reference standard for its diagnosis. However, a clear definition of AVWS and its severity or quantitative evaluation methods for VWF multimer analysis have yet to be established [4]. On the other hand, studies have shown that the loss of HMWMs leads to a disproportionate decrease in qualitative platelet-related activity assays of VWF, such as VWF:RCo (ristocetin cofactor activity, which reflects VWF–platelet interactions) and VWF:CB (collagen binding activity, which reflects VWF–connective tissue interactions), compared with the level of VWF antigen (VWF:Ag) [5]. Consequently, the VWF:RCo/VWF:Ag and VWF:CB/VWF:Ag ratios are <0.7, mirroring the pattern observed in type 2A von Willebrand disease (VWD) [6,7], and could serve as routine screening tools to evaluate VWF function and identify the loss of VWF large multimers [8].

The clinical manifestation of AVWS is highly variable. In the majority of cases, patients are asymptomatic; however, in others, symptoms can range from bleeding to thrombosis and nonphysiological angiogenesis. Among the various forms of cardiovascular disease, AVWS has been extensively documented in the context of valvular heart disease, particularly in cases of aortic stenosis [9], mitral regurgitation [10], mechanical circulatory support, either extracorporeal membrane oxygenation or left ventricular assist device [11]. With regard to congenital heart diseases, AVWS has been described in conditions such as hypertrophic obstructive cardiomyopathy, tetralogy of Fallot, or pulmonary hypertension, particularly among patients with Eisenmenger syndrome [12,13]. Furthermore, its presentation was elegantly demonstrated in infants undergoing surgical intervention [14]. On the other hand, relatively common congenital lesions, such as patent ductus arteriosus (PDA) and ventricular septal defect (VSD), can also create hemodynamic conditions that increase susceptibility to AVWS. The pathological blood shunting between the high-pressure systemic and low-pressure pulmonary circulations induces hemodynamic states that elevate shear stress. Consequently, this can lead to the loss of VWF HMWMs or reveal an underlying qualitative defect. However, to the best of our knowledge, evidence on the prevalence of AVWS in the pediatric population with PDA and VSD is limited and inconsistent [14], primarily derived from case reports and small observational studies [[15], [16], [17]].

We investigated whether young children with isolated congenital heart diseases, such as PDA and VSD, exhibit qualitative disturbances in platelet-related activity and whether this translates to the loss of HMWMs, consequently leading to the occurrence of AVWS-like phenotype. Identifying potential bleeding-prone conditions at such a young age is crucial, as it can prevent serious bleeding complications later on.

## 2 Methods

### 2.1 Study design

**Figure 1:**
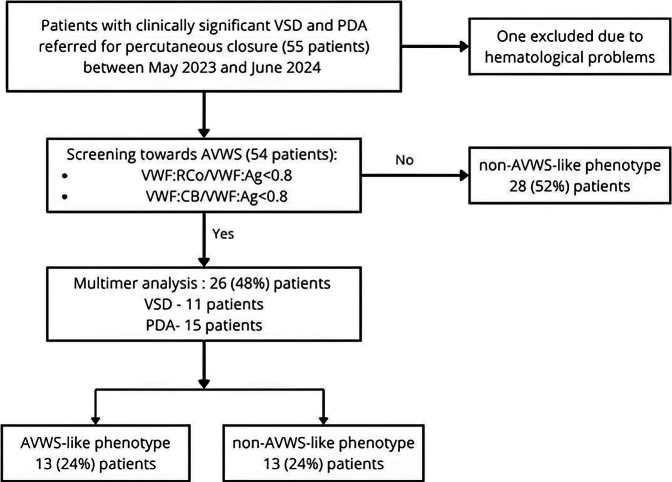
Study flow chart. AVWS, acquired von Willebrand syndrome; PDA, patent ductus arteriosus; VSD, ventricular septal defect; VWF:Ag, von Willebrand factor antigen level; VWF:CB, von Willebrand factor collagen binding activity; VWF:RCo, von Willebrand factor ristocetin cofactor activity.

We conducted a single center, cross-sectional study to evaluate the clinical and laboratory manifestations of AVWS in children with congenital heart disease, specifically PDA and VSD. The study included patients who underwent percutaneous interventional correction of these conditions at our hospital between May 2023 and June 2024. The study was approved by the local ethics committee (47/2023/B) and conducted in compliance with the Declaration of Helsinki. Written informed consent was obtained from the parents of all participating children prior to inclusion and before any study-related procedures were carried out. The study flow chart is illustrated in Figure 1.

### 2.2 Patient selection

We included all patients aged 0 to 18 years with a body weight >6 kg who had hemodynamically and/or clinically significant PDA and VSD and were referred for percutaneous closure of the defects. Patients with a history of hemorrhagic diathesis, those on antiplatelet or anticoagulation therapy, individuals with elevated C-reactive protein levels upon admission suggesting an active inflammatory process, and children with concurrent hypothyroidism or neoplastic diseases were excluded from the study.

Blood sampling and echocardiographic assessment were performed the day before the index procedure upon the patient’s admission to the hospital. All echocardiograms were performed by 2 pediatric cardiologists (WT, PW). In addition to standard echocardiographic measurements, in VSD patients, the pressure gradient between the left and right ventricles was calculated using the modified Bernoulli equation. This calculation utilized Doppler measurements of the highest peak systolic instantaneous gradient at any site. In patients with PDA, the pressure gradient between the aorta and the pulmonary artery was measured invasively during percutaneous closure of the defect by evaluating the difference between the systolic pressure in the ascending aorta and that in the main pulmonary artery.

The following clinical data were collected: transthoracic echocardiography findings, laboratory results, details of blood component therapy, history of bleeding and thrombotic events (including any need for blood product transfusion), and family history of bleeding disorders.

### 2.3 Laboratory analysis

Venous blood samples were drawn into 3.2% (0.109 mol/L) sodium citrate tubes (1 part sodium citrate to 9 parts venous blood), centrifuged at 2000 × *g* for 10 minutes within 30 minutes of drawing, and stored in aliquots at −80 °C for further analysis.

Complete blood counts and basic coagulation tests (prothrombin time, activated partial thromboplastin time, and fibrinogen) were determined using routine laboratory assays.

**Figure 2:**
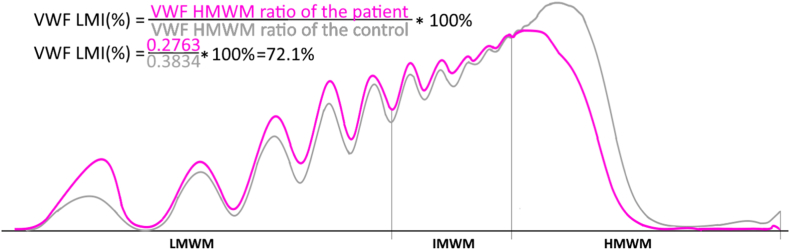
Comparison of VWF multimer patterns between normal plasma control and a patient with an AVWS-like phenotype. The pink line represents the VWF multimer distribution in the plasma of a patient with an AVWS-like phenotype, while the gray line corresponds to normal control plasma. Multimer distribution is expressed as the mean percentage of the area under the curve (%AUC) for low-, intermediate-, and high-molecular-weight multimers. The formula used to calculate the VWF-LMI is also provided. An illustrative example is shown in this figure. AVWS, acquired von Willebrand syndrome; HMWM, high-molecular-weight multimer; IMWM, intermediate-molecular weight multimer; LMI, large multimer index; LMWM, low-molecular-weight multimer; VWF, von Willebrand factor.

Factor (F)VIII activity was measured using a coagulometric assay with FVIII-deficient plasma (Siemens). The activity (ristocetin cofactor VWF:RCo) and level of VWF (VWF:Ag) were determined using immunoturbidimetric methods (BC von Willebrand Reagent and VWF:Ag; Siemens). The binding of VWF to collagen (VWF:CB) was performed using a chemiluminescent method (Acustar VWF:CB; Werfen). VWF:CB/VWF:Ag and VWF:RCo /VWF:Ag ratios were calculated. VWF:RCo/VWF:Ag and/or VWF:CB/VWF:Ag ratios <0.7 serve as markers of impaired VWF function and are widely used as indicators of AVWS. However, to determine qualitative VWF defects and the loss of HMWMs, this study utilized VWF:RCo/VWF:Ag and/or VWF:CB/VWF:Ag ratios <0.8 to mitigate the risk of overlooking an AVWS diagnosis, as along with high specificity, these ratios exhibit low sensitivity [4,15]. VWF multimers were analyzed on a Hydrasys 2 instrument (Sebia) with a ready-to-use sodium dodecyl sulfate agarose gel (Hydragel 11 von Willebrand multimers; Sebia). Multimers were visualized directly on the gel (without protein transfer) according to the manufacturer’s instructions. Curves were produced using the manufacturer’s gel scanner and interpretation software. Normal plasma with VWF:Ag concentrations >100% were used as a control. Based on densitometric analysis for quantitative evaluation, VWF multimer bands from the lowest to the fifth, from the sixth to the tenth, and higher than the tenth were classified as low-molecular-weight multimers (LMWMs), intermediate-molecular-weight multimers (IMWMs), and HMWMs, respectively (Figure 2). The above categorization was selected after a thorough review of existing research on AVWS related to cardiovascular conditions. Notably, a Japanese research team studying AVWS in cardiovascular diseases employed the same classification system. In their studies [12,18], multimers were classified as LMWMs (bands 1-5), IMWMs (bands 6-10), and HMWMs (bands >10). Similarly, Stockschlaeder et al. [3] defined small, intermediate, and large multimers using the same criteria. Icheva et al. also adopted this classification approach in their studies of AVWS in infants with congenital heart disease [14] and during congenital heart surgery [15]. Due to its consistent application across studies, particularly in research on AVWS related to cardiovascular conditions, this multimer classification enhances comparability with existing research and increases the clinical relevance of our results. The VWF large multimer index (VWF-LMI), which has been widely used recently [18,19], was calculated to provide a quantitative assessment of HMWM loss. The index was calculated as the ratio of the patient’s VWF large multimer ratio to that of the control, which is analyzed in the same gel (Figure 2). An AVWS-like phenotype was defined as a VWF:RCo/VWF:Ag ratio and/or a VWF:CB/VWF:Ag ratio of <0.8, along with a simultaneous relative reduction in VWF HMWMs, resulting in a disproportion between the HMWMs and LMWMs.

### 2.4 Statistical analysis

Statistical analysis was conducted using R software version 4.4.2 (R Development Core Team). Continuous variables are presented as mean ± SD for normally or log-normally distributed data and as median and IQR otherwise. Differences were compared with a Student’s *t*-test or Mann–Whitney *U*-test, depending on the distribution. Categorical variables are presented as numbers (percentages), and their differences were assessed with Pearson’s chi-squared or Fisher’s exact test. The normality of all tested distributions was assessed with the Shapiro–Wilk test. Relationships between selected continuous variables were analyzed using Pearson correlations with Fisher’s *z*-test. Predictive power of analyzed parameters was assessed by computing area under receiver operating characteristic curve (ROC-AUC) with 95% bootstrap CIs, as well as optimal cutoff point maximizing Youden index. For each such cutoff point, it was also checked (using Fisher’s test) if the resulting classes differed significantly in AVWS prevalence, and what the resulting specificity and sensitivity were. R packages pROC [20] and cutpointr [21] were used for AUC calculation and cutoff point selection, respectively. *P* < .05 was considered statistically significant for all tests applied in the analysis.

## 3 Results

A total of 55 consecutive patients who underwent transcatheter PDA or VSD closure between May 2023 and June 2024 were enrolled in the study. One patient had to be excluded from the data analysis due to the detection of a congenital bleeding disorder. Of the remaining 54 patients, 20 were admitted for VSD closure and 34 for PDA closure. Detailed patient characteristics and baseline laboratory parameters, including coagulation parameters, are presented in Table 1. Overall, the 2 groups showed no differences in terms of age, sex, weight, or prematurity. Laboratory tests, including coagulation parameters, revealed no significant differences between the PDA and VSD groups, except for a slightly higher platelet count and VWF:CB/VWF:Ag ratio, along with lower fibrinogen level in the former group. The significant gradient differences between the 2 groups (63 [58-69] vs 80 [69.5-89.5], *P* < .001) were likely due to the differing measurement techniques: an invasive method was used for PDA patients, while an echocardiographic approach was adopted for VSD patients.

**Table 1:** Patient demographics and baseline characteristics.

Variables	PDA group	VSD group	Total	*P* value
Age, y	2.8 (1.7-6.6)	2.8 (2.4-6.2)	2.8 (1.8-6.5)	.60
Male, *n* (%)	10 (18.5)	9 (16.7)	19 (35.2)	.39
Weight, kg	16.1 ± 7.7	16.9 ± 6.6	16.4 ± 7.3	.43
Premature, *n* (%)	6 (11.1)	2 (3.7)	8 (14.8)	.70
**Complete blood count analysis**				
RBC, ×10^6^/μL	4.44 ± 0.32	4.39 ± 0.28	4.42 ± 0.3	.58
Hemoglobin, mg/dL	12.3 (11.8-12.9)	12.1 (11.9-12.4)	12.2 (11.8-12.7)	.22
Hematocrit, %	34.9 (33.9-36.4)	34.4 (33.4-36.0)	34.7 (33.7-36.3)	.24
WBC, ×10^3^/μL	8.3 ± 2.9	7.5 ± 2.1	8 ± 2.7	.30
Platelets, ×10^3^/μL	346.4 ± 85.7	292.1 ± 91.8	326.3 ± 91.1	.03
**Coagulation parameters**				
aPTT, s	30.2 (28.4-32.9)	30.9 (27.0-32.1)	30.5 (28.1-32.9)	.51
PT (INR)	0.97 ± 0.1	0.98 ± 0.1	0.97 ± 0.1	.56
Fibrinogen, mg/dL	2.3 ± 0.5	2.6 ± 0.5	2.4 ± 0.5	.03
Factor VIII, %	77.0 ± 25.0	94.3 ± 37.5	83.4 ± 31.1	.08
VWF:Ag, %	82.4 ± 22.6	99.0 ± 43.3	88.5 ± 32.5	.19
VWF:RCo, %	70.4 ± 28.1	81.9 ± 54.7	74.7 ± 40.0	.60
VWF:CB, %	77.8 ± 21.5	86.0 ± 44.6	80.8 ± 31.6	.89
VWF:RCo/VWF:Ag	0.85 ± 0.21	0.79 ± 0.19	0.83 ± 0.21	.29
VWF:CB/VWF:Ag	0.96 (0.9-1)	0.9 (0.83-0.93)	0.94 (0.87-0.99)	.006

Data are median (IQR), mean ± SD, or frequency (percentage).

aPTT, activated partial thromboplastin time; INR, international normalized ratio; PDA, patent ductus arteriosus; PT, prothrombin time; RBC, red blood cells; VSD, ventricular septal defect; VWF:Ag, von Willebrand factor antigen level; VWF:CB, von Willebrand factor collagen binding activity; VWF:RCo, von Willebrand factor ristocetin cofactor activity; WBC, white blood cells.

To identify patients with an AVWS-like phenotype, screening for HMWM loss and VWF quality defects was performed by assessing the VWF:RCo/VWF:Ag and VWF:CB/VWF:Ag ratios, with a threshold <0.8. Of the study cohort, 26 (48.1%) patients met the above criteria, including 15 (58%) patients with PDA and 11 (42%) patients with VSD (*P* = .2). Densitometric gel analysis of VWF multimers in the aforementioned patients who met the screening criteria for the AVWS-like phenotype showed that half of them, namely 13 children (24.1% of all patients included in the study), had significantly reduced HMWM levels (27.3 ± 2.9% vs 38.8 ± 5.5%; *P* < .001) and fulfilled the diagnostic criteria for AVWS-like phenotype. In essence, there were no differences between the 2 groups of patients regarding age, sex, prematurity, weight, complete blood count analysis, or lesion type (data not shown). Additionally, the initial pressure gradient across the lesion was similar between patients with an AVWS-like phenotype and those without (70.5 [65-75.3] vs 66 [61-79] mmHg; *P* = .47). Numerically, the hemoglobin level was slightly lower in the AVWS-positive group, showing borderline significance (11.9 ± 0.97 vs 12.4 ± 0.6 mg/dL, *P* = .05), yet it did not qualify as anemia. Among the coagulation parameters, both patient groups were homogeneous, except for the VWF:RCo/VWF:Ag and VWF:CB/VWF:Ag ratios, which were used as screening criteria (Table 2).

**Table 2:** Laboratory parameters according to AVWS phenotype status.

Laboratory parameters	AVWS-like phenotype *n* = 13 (24.1%)	Non-AVWS-like phenotype *n* = 41 (75.9%)	*P* value
aPTT, s	30.2 (28-32.7)	30.2 (28-32.7)	.38
INR	0.99 (0.9-1.0)	0.96 (0.9-1.0)	.4
Fibrinogen, mg/dL	2.4 ± 0.5	2.45 ± 0.5	.52
Platelets, ×10^3^/μL	318 ± 99.4	328.9 ± 89.4	.71
Hemoglobin, mg/dL	11.9 ± 0.97	12.35 ± 0.6	.05
Factor VIII, %	64.3 (54.6-111)	83.2 (68.2-102.4)	.27
VWF:Ag, %	96.6 (60.5-106.7)	79.8 (69.9-95.5)	.83
VWF:RCo, %	64.0 ± 30.4	78.0 ± 42.3	.22
VWF:CB, %	69.1 ± 23.8	84.6 ± 33.1	.10
VWF:RCo/VWF:Ag	0.68 (0.65-0.74)	0.81 (0.74-0.96)	.002
VWF:CB/VWF:Ag	0.84 (0.78-0.9)	0.96 (0.91-0.99)	<.001

Data are median (IQR), mean ± SD, or frequency (%).

aPTT, activated partial thromboplastin time; AVWS, acquired von Willebrand syndrome; INR, international normalized ratio; VWF:Ag, von Willebrand factor antigen level; VWF:CB, von Willebrand factor collagen binding activity; VWF:RCo, von Willebrand factor ristocetin cofactor activity.

**Figure 3:**
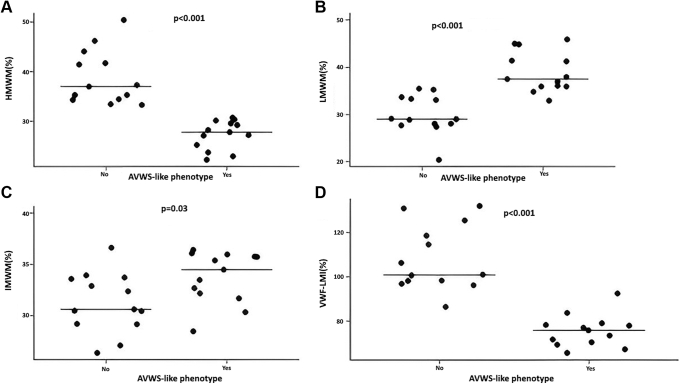
The distribution of multimers (A–C) and VWF-LMI (D) among patients with AVWS-like and non-AVWS-like phenotypes. Multimer analysis revealed that patients with AVWS-like phenotype showed a significant reduction in HMWMs and VWF-LMI, while IMWM and LMWM levels were lower compared with patients without non-AVWS-like phenotype. AVWS, acquired von Willebrand syndrome; HMWM, high-molecular-weight multimer; IMWM, intermediate-molecular-weight multimer; LMI, large multimer index; LMWM, low-molecular-weight multimer; VWF, von Willebrand factor.

**Figure 4:**
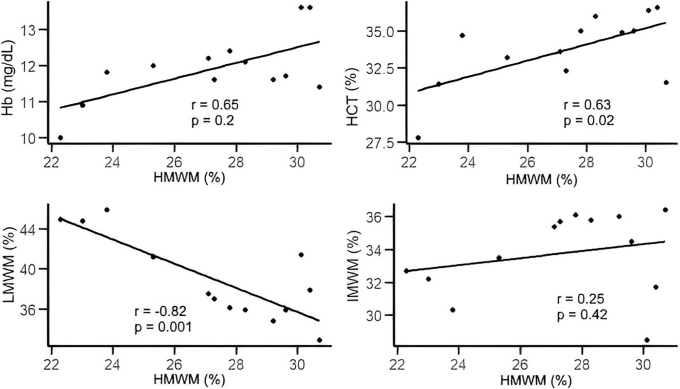
Association between HMWM levels and various laboratory parameters. HMWM levels showed a moderate positive correlation with Hb and HCT levels and a strong negative correlation with LMWM levels. Hb, hemoglobin; HCT, hematocrit; HMWM, high-molecular-weight multimer; LMWM, low-molecular-weight multimer.

The distribution of VWF multimers revealed significant differences between patients with AVWS-like and non-AVWS-like phenotypes. Notably, children exhibiting an AVWS-like phenotype had a lower percentage of HMWMs (27.3 ± 2.9% vs 38.8 ± 5.5%, *P* < .001), while IMWMs and LMWMs were increased (33.8 ± 2.5% vs 31.3 ± 2.9%, *P* = .03 and 39.0 ± 4.3% vs 30.0 ± 4.2%, *P* < .001, respectively; Figure 3). In this group of children, HMWM levels demonstrated a moderate positive correlation with erythrocyte count, hematocrit, and hemoglobin levels as well as a strong negative correlation with LMWM levels (Figure 4). Furthermore, no correlation was observed between HMWM levels and the pressure gradient in any patient group—including the VSD and PDA subgroups—or with platelet count or fibrinogen levels (data not shown). In quantitative multimer analysis, VWF-LMI was significantly lower in patients with an AVWS-like phenotype compared to those with a non-AVWS phenotype (75.5 ± 7.3 vs 108.1 ± 14.7, *P* < .001), as shown in Figure 3D.

**Figure 5:**
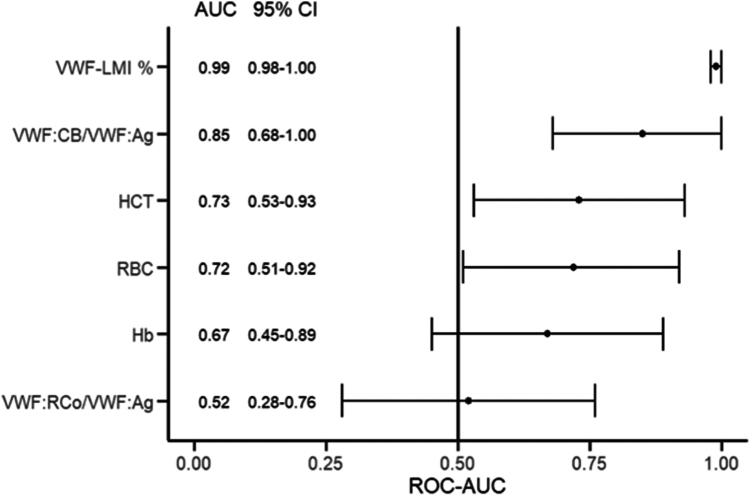
Analysis of possible predictors for an AVWS-like phenotype. Alongside VWF-LMI, the VWF:CB/VWF:Ag ratio showed the best predictability compared with the other investigated parameters. AUC, area under the curve; AVWS, acquired von Willebrand syndrome; Hb, hemoglobin; HCT, hematocrit; RBC, red blood cell; ROC-AUC, area under the receiver operating characteristic curve; VWF, von Willebrand factor; VWF:Ag, VWF antigen level; VWF:CB, VWF collagen binding activity; VWF-LMI, von Willebrand factor large multimer index; VWF:RCo, VWF ristocetin cofactor activity.

Various diagnostic factors were evaluated for their accuracy in predicting AVWS using receiver operating characteristic curve analysis. Among them, the VWF-LMI provided the best predictive power (AUC, 0.99; 95% CI, 0.98-1.0; *P* < .001). When the threshold was set at <0.8, the sensitivity and specificity for the loss of large multimers were 1.0 and 0.87, respectively. In comparison, the accuracy of the VWF:CB/VWF:Ag ratio was poorer (AUC, 0.85; 95% CI, 0.68-1.0; *P* = .002). When the threshold was set at VWF:CB/VWF:Ag ratio <0.8, the sensitivity and specificity for the loss of large multimers were 0.57 and 0.80, respectively (Figure 5).

The study group exhibited no prior bleeding history. Furthermore, all percutaneous defect closures for both PDA and VSD were completed without any significant bleeding events.

## 4 Discussion

Our data indicate that an AVWS-like phenotype is present in almost one-quarter of young pediatric patients with isolated VSD and PDA. Although these 2 lesions differ in pathophysiology, they share a common mechanism that leads to AVWS. Specifically, in both congenital defects, blood is shunted from the high-pressure systemic circulation to the low-pressure pulmonary circulation through either a PDA [22] or a VSD. This creates high shear stress in the circulation, which enhances secretion, unfolding [15], and proteolytic clearance of HMWMs by ADAMTS-13 [23], resulting in a decrease or loss of the latter. In a previous study, Rauch et al. [16] reported a 33% incidence of AVWS in a small cohort of 12 patients with PDA, a rate slightly higher than that observed in our population. In contrast, Icheva et al. [15] found no association between PDA and AVWS in neonates in their case series study. They hypothesized that the development of AVWS may take a variable amount of time and may manifest at a later stage. At the same time, the prevalence of AVWS in isolated VSD cases remains uncertain, as most research has focused on patients with complex congenital defects that include VSD [13,17], leaving limited data on isolated cases [24]. Although the 2 cardiac defects share similar pathophysiological features, their underlying mechanisms vary slightly. This variation may partly account for the differences in platelet counts, even though they remain within the normal range. The slightly lower platelet count in the VSD group may be attributed to a potential association with thrombocytopenia, possibly through an immunological mechanism or increased platelet activation. From a pathophysiological perspective, VSD involves a systolic, high-velocity left-to-right shunt, while PDA typically features a continuous left-to-right flow, producing a distinctive continuous “machine-like” murmur. Consequently, it is plausible that shear stress in VSD defects, which causes stretching and rupture of VWF multimers and diminishes their function, is higher than in PDA defects. Nonetheless, these findings may warrant confirmation in larger patient groups.

The reference standard for the diagnosis of AVWS is the time-consuming VWF multimer analysis. However, no clear definition of AVWS or its severity classification exists because quantitative assessment methods for VWF multimer analysis have yet to be established [4].

For screening purposes to identify impaired VWF function as indicators of AVWS, we adjusted the VWF:RCo/VWF:Ag and/or VWF:CB/VWF:Ag ratios to be <0.8. Consequently, 26 (48.1%) patients met these criteria and underwent VWF multimer analysis, resulting in a subsequent diagnosis of an AVWS-like phenotype in 13 (24.1%) participants. Interestingly, both groups in our study exhibited comparable pressure gradients across the lesion (70.5 [65-75.3] vs 66 [61-79] mmHg, *P* = .47), with no correlation found between HMWM loss and the gradient across the lesion. Our data differ from previous studies that showed a strong association between increased maximal pressure gradients across the aortic valve, HMWM loss, and the development of AVWS in patients with aortic stenosis [9,18]. Indeed, to evaluate how varying pressure levels affect AVWS occurrence in patients with PDA and VSD, it is essential to compare patients with different gradients across the lesion, similar to the approach used in aortic stenosis cases. However, our cohort was quite homogeneous, and there were no significant high or low-pressure extremes. Unlike aortic stenosis, a higher gradient in the case of PDA or VSD does not indicate disease progression. Furthermore, as the disease advances, higher pulmonary pressures emerge, resulting in lower gradients and velocities through the shunt. Notably, our patients were all young children without pulmonary hypertension, limiting our ability to investigate the impact of gradient on HMWM loss. Our results align with previous studies showing that the simple presence of a PDA is strongly, but not necessarily, associated with AVWS [16].

Patients with AVWS-like and non-AVWS-like phenotypes did not differ in FVIII, VWF:Ag, VWF:RCo, or VWF:CB, which, according to the available literature, may or may not be slightly reduced in the AVWS-positive individuals [25]. Furthermore, according to the American Society of Hematology and the International Society on Thrombosis and Haemostasis, a decrease in VWF:RCo, VWF:CB, and/or VWF:Ag are essential criteria for the diagnosis of hereditary VWD [7], whereas they are not included in the diagnosis of AVWS. Of note, the recent paper on AVWS in aortic stenosis patients revealed relatively high levels of VWF:Ag and VWF:RCo, both exceeding 100%, although the latter was statistically significantly lower compared with the control group [4].

As expected, patients with an AVWS-like phenotype exhibited lower VWF:RCo/VWF:Ag and VWF:CB/VWF:Ag ratios, with the former consistently falling <0.7. Our findings align with earlier studies indicating that a VWF:RCo/VWF:Ag ratio <0.7 is common among patients with AVWS. Icheva et al. noted its extremely high specificity, reaching 100%, though its sensitivity remains low at 36%, suggesting it could serve as a marker for AVWS [15]. Our data further support that a significant reduction in VWF–platelet (eg, VWF:RCo) or VWF–connective tissue (eg, VWF:CB) interactions compared with VWF:Ag is characteristic of the loss of large multimers in AVWS [5,26].

In a densitometric multimer analysis of 26 pediatric patients (48.1%), a strong negative correlation was observed between HMWMs and LMWMs in the AVWS-like phenotype group. Therefore, it can be deduced that in patients with VSD and PDA exhibiting an AVWS-like phenotype the relative decline in HMWMs is more likely linked to an increase in the LMWM pool rather than the IMWM pool. This observation is consistent with the findings of Goldfarb et al. [11], who reported that all patients with AVWS exhibited high normal or elevated levels of LMWMs. Indeed, the distribution of multimers indicates the balance between multimer assembly, their clearance from the bloodstream, and proteolysis by ADAMTS-13 [5]. In individuals with AVWS, a significant relative deficiency of HMWMs results from increased cleavage by ADAMTS-13 under conditions of higher shear stress [4]. The mechanism described in AVWS patients, involving multimer redistribution toward LMWMs, may resemble VWD type 2B phenotype. In this scenario, HMWMs bind spontaneously to platelets after secretion and are subsequently cleaved by ADAMTS-13. The resulting small multimers are ineffective in facilitating platelet adhesion and appear to bind platelets, thereby hindering their interaction with connective tissue directly [5,27].

Similar to patients with severe aortic stenosis, our cohort with an AVWS-like phenotype exhibited a significantly reduced VWF-LMI (75.5 ± 7.3 vs 108.1 ± 14.7; *P* < .001), falling <0.8 threshold established by Tamura et al. [18]. Among the evaluated parameters, VWF-LMI was the most reliable predictor of AVWS-related loss of large multimers, demonstrating a sensitivity of 1.0 and specificity of 0.87. Additionally, the VWF:CB/VWF:Ag ratio was the only other marker able to predict HMWM loss when <0.8, with a sensitivity of 0.57 and specificity of 0.80, while a VWF:RCo/VWF:Ag ratio <0.8 failed to predict AVWS occurrence in our patient group. The rationale for the disproportionate decrease in VWF–connective tissue interactions (eg, VWF:CB) compared with VWF–platelet interactions (eg, VWF:RCo) relative to VWF:Ag remains incompletely understood. This discrepancy is likely due to differences in assay performance. Specifically, the VWF:RCo assay is known to be more variable and less sensitive to HMWM loss, while the AcuStar-based VWF:CB assay demonstrates greater sensitivity and reproducibility [28,29]. Nevertheless, further research is necessary to address these issues and provide a more comprehensive understanding.

The bleeding rates observed during the procedure were minimal across the entire cohort, with no significant increase recorded in patients with an AVWS-like phenotype. This finding aligns with previous observations, which suggest that AVWS does not lead to excessive bleeding in the absence of open wound surfaces but primarily after major trauma and surgery [14,25,26]. Furthermore, the symptoms of bleeding can vary, with the most common manifestations including mucocutaneous bleeding, heavy menstrual bleeding, and easy bruising. Given that the median age of our patients is 2.8 years, it is probable that they have not yet encountered these symptoms [30].

This study has several notable limitations. First, the sample size was relatively small. Second, it was conducted at a single center, which may have introduced bias. Third, blood samples were collected only before the lesion closure, leaving a gap in data regarding patients’ AVWS status postintervention. Additionally, only patients who met the screening criteria for HMWM loss underwent the multimer analysis assay. Therefore, it is possible that some patients with an AVWS-like phenotype were overlooked. Finally, we cannot rule out the possibility of unknown confounding factors that could impact patients’ AVWS status.

To summarize, an AVWS-like phenotype is relatively common, affecting nearly a quarter of young children with isolated VSD and PDA, yet it remains clinically silent. In addition to VWF multimer analysis and VWF-LMI assessment, the VWF:CB/VWF:Ag ratio appears to be suitable for screening diagnosis in this cohort and could be incorporated into the routine diagnostic work-up of the complex prior to surgical or interventional procedures. Although no bleeding complications were observed during the study, the impact of an AVWS-like phenotype on periprocedural bleeding risk in these children remains uncertain. It is important to note that the study population was relatively young, and bleeding complications may emerge later on.
